# Supplementary material for: Modeling glioblastoma heterogeneity as a dynamic network of cell states
Source: Mol Syst Biol. 2021 Sep 16;17(9):e10105. doi: 10.15252/msb.202010105 (PMC8444284; doi:10.15252/msb.202010105)
Supplement: Supplementary file 6 — Source Data for Figure 5 [file MSB-17-e10105-s004.zip › Figure5A_sourcedata/GSEA_3017/nosigdb_stateD.GseaPreranked.1622795010397/DOROSZKO_U3054MG_CLUSTER_0.html]

Details for gene set DOROSZKO\_U3054MG\_CLUSTER\_0[GSEA]

|  || Dataset | state23017 |
| Phenotype | NoPhenotypeAvailable |
| Upregulated in class | na\_pos |
| GeneSet | DOROSZKO\_U3054MG\_CLUSTER\_0 |
| Enrichment Score (ES) | 0.18243687 |
| Normalized Enrichment Score (NES) | 0.9732978 |
| Nominal p-value | 0.43979058 |
| FDR q-value | 0.605157 |
| FWER p-Value | 0.991 |
Table: GSEA Results Summary

  

Fig 1: Enrichment plot: DOROSZKO\_U3054MG\_CLUSTER\_0      
 Profile of the Running ES Score & Positions of GeneSet Members on the Rank Ordered List

  

| PROBE | GENE SYMBOL | GENE\_TITLE | RANK IN GENE LIST | RANK METRIC SCORE | RUNNING ES | CORE ENRICHMENT || 1 | FABP5 |  |  | 1 | 0.885 | 0.1188 | Yes |
| 2 | OLIG1 |  |  | 10 | 0.517 | 0.1824 | Yes |
| 3 | CPM |  |  | 289 | -0.263 | 0.0044 | No |
| 4 | COL4A5 |  |  | 470 | -0.282 | -0.0958 | No |
| 5 | CKB |  |  | 554 | -0.292 | -0.1201 | No |
| 6 | PPP2R2B |  |  | 589 | -0.297 | -0.1061 | No |
| 7 | TSC22D4 |  |  | 602 | -0.301 | -0.0747 | No |
| 8 | SORL1 |  |  | 652 | -0.311 | -0.0704 | No |
| 9 | PPP1R9A |  |  | 675 | -0.315 | -0.0448 | No |
| 10 | GPR37L1 |  |  | 683 | -0.315 | -0.0076 | No |
| 11 | TRIM2 |  |  | 759 | -0.333 | -0.0202 | No |
| 12 | SLC44A1 |  |  | 814 | -0.348 | -0.0146 | No |
| 13 | PMEPA1 |  |  | 868 | -0.363 | -0.0063 | No |
| 14 | GPC4 |  |  | 891 | -0.369 | 0.0267 | No |
| 15 | SAP30 |  |  | 905 | -0.376 | 0.0675 | No |
| 16 | CDH2 |  |  | 1007 | -0.418 | 0.0464 | No |
| 17 | NCAM1 |  |  | 1049 | -0.445 | 0.0750 | No |
| 18 | CADM1 |  |  | 1103 | -0.469 | 0.0977 | No |
| 19 | TTYH1 |  |  | 1144 | -0.505 | 0.1352 | No |
Table: GSEA details [plain text format]

  

Fig 2: DOROSZKO\_U3054MG\_CLUSTER\_0: Random ES distribution      
 Gene set null distribution of ES for **DOROSZKO\_U3054MG\_CLUSTER\_0**
